# Pilomyxoid Astrocytoma Presenting With Developmental Regression: A Case Report

**DOI:** 10.7759/cureus.67167

**Published:** 2024-08-19

**Authors:** Om Prasanth Reddy Avuthu, Shradha Salunkhe, Manojkumar G Patil, Archana C. Buch, Shailaja V Mane, Ashish Chugh

**Affiliations:** 1 Paediatrics, Dr. D. Y. Patil Medical College, Hospital & Research Centre, Dr. D. Y. Patil Vidyapeeth (Deemed to be University), Pune, IND; 2 Pathology, Dr. D. Y. Patil Medical College, Hospital & Research Centre, Dr. D. Y. Patil Vidyapeeth (Deemed to be University), Pune, IND; 3 Neurosurgery, Dr. D. Y. Patil Medical College, Hospital & Research Centre, Dr. D. Y. Patil Vidyapeeth (Deemed to be University), Pune, IND

**Keywords:** pilomyxoid astrocytoma, pediatrics and neonatology, neuro oncology, low grade gliomas, pediatric solid tumours

## Abstract

Pilomyxoid astrocytoma (PMA) is a subtype of pilocytic astrocytoma (PA). PMA tends to exhibit a more aggressive course compared to PA. We present a case of a two-year-old male with a PMA in the suprasellar region who presented with developmental regression, loss of previously attained milestones such as the ability to hold his neck, walk, and talk, along with hypotonia in all four limbs. Serum cortisol and thyroid-stimulating hormone (TSH) levels were measured to rule out endocrine disturbances and were within normal limits. Magnetic resonance imaging (MRI) of the brain showed a solid lesion in the suprasellar region, extending into the pituitary and interpeduncular fossae, compressing the pituitary gland, and effacing the third ventricle, causing cerebrospinal fluid (CSF) flow obstruction and lateral ventricle dilation. The tumor appears hypointense on T1 and hyperintense on T2, with fluid-attenuated inversion recovery (FLAIR), peripheral contrast enhancement, and no calcification, consistent with PMA. The CSF analysis was negative for malignant cells. Histopathological examination revealed monomorphous bipolar and spindle cells in an angiocentric pattern with a myxoid background, without rosenthal fibers, mitoses, or eosinophilic granular bodies, consistent with PMA but not seen in PA. Immunohistochemistry showed strong positivity for glial fibrillary acidic protein (GFAP) and S100, with a Ki-67 index of 3-4%, indicating a low-grade tumor. The preferred treatment is surgical resection, but due to the tumor's deep location and potential long-term neurological effects, the parents opted against surgery. A ventriculoperitoneal shunt was placed to alleviate CSF flow, following which the child showed mild improvement in symptoms. Treatment of nonresectable astrocytomas was controversial, but gross total surgical resection offers better disease control. Chemotherapy is for patients with recurrence or where total resection of the tumor is not possible, and radiotherapy, though the long-term disease control is good, has a variable visual outcome.

## Introduction

In India, central nervous system (CNS) tumors occur at a rate of five to 10 cases per 100,000 individuals, comprising approximately 2% of all malignant neoplasms. The most common primary tumor was astrocytoma, comprising 38.7% of cases. Most astrocytic tumors reported were low-grade, predominantly pilocytic astrocytomas (PA) and subependymal giant cell astrocytomas [1]. PA are typically observed in children. PA are slow-growing tumors with a good prognosis and are classified as Grade I according to the previous World Health Organization (WHO) classification. In contrast, pilomyxoid astrocytomas (PMA), considered a variant of PA, exhibit a more aggressive clinical course and a higher recurrence rate. They preferentially occur in the optic chiasmatic/hypothalamic region and are classified as Grade II tumors as per the previous WHO classification [2], whereas the recent classification kept them under pediatric-type diffuse low-grade gliomas [3]. Here, we discuss the case of a two-year-old male child diagnosed with PMA, presenting with regression of developmental milestones.

## Case presentation

**Figure 1:**
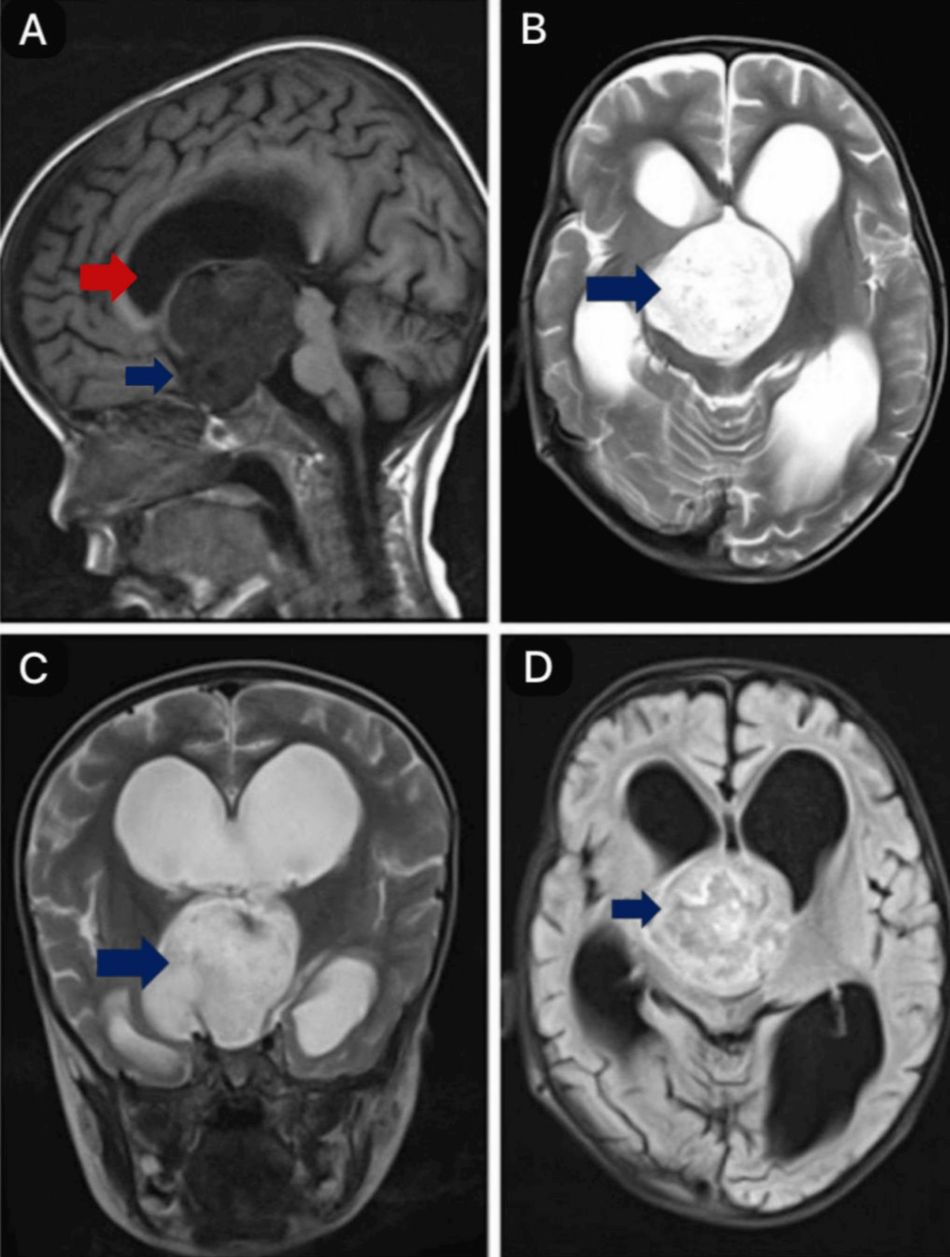
MRI brain A) A T1-weighted sagittal section image shows a heterogeneously hypointense tumor causing effacement of the 3rd ventricle (indicated by a blue arrow), causing proximal obstructive dilatation of the lateral ventricle (indicated by a red arrow). B and C) T2-weighted axial and coronal sections show heterogeneously hyperintense tumors in the suprasellar region (indicated by a blue arrow). D) FLAIR image showing a heterogeneously hyperintense tumor with central/cystic areas showing suppression (indicated by a blue arrow). MRI: magnetic resonance imaging; FLAIR: fluid-attenuated inversion recovery

**Figure 2:**
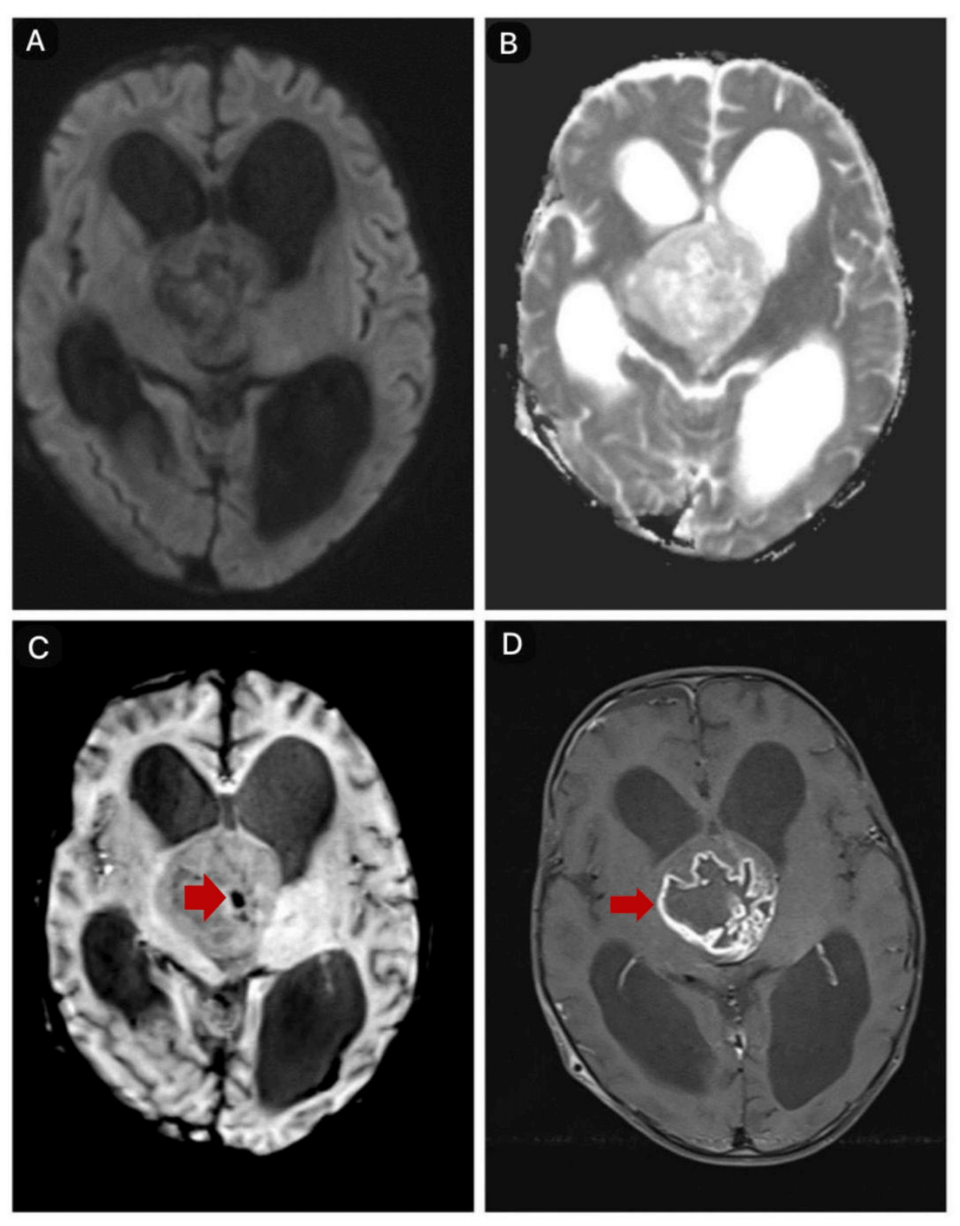
MRI brain A and B) Diffusion-weighted imaging showing no diffusion restriction and corresponding ADC image. C) Susceptibility-weighted imaging showing a signal dropout (indicated by a red arrow) suggestive of hemorrhage. D) T1 fat-saturated post-contrast image showing heterogeneous peripheral enhancement (indicated by a red arrow). MRI: magnetic resonance imaging; ADC: apparent diffusion coefficient

A two-year-old male child, born from a non-consanguineous marriage with no significant medical history, presented with sudden loss of neck control, inability to support his neck, inability to sit or stand without support, decreased speech output, and normal sensorium. He had previously reached all developmental milestones appropriate for his age and was able to speak clearly. During examination, the child appeared conscious but irritable, with a downward gaze, brisk reflexes, and hypotonia in all four limbs. An ophthalmologic evaluation was normal. The child was started on mannitol in view of the raised intracranial pressure. A magnetic resonance imaging (MRI) scan revealed a well-defined, solid, heterogeneously enhancing lesion in the suprasellar region. The optic chiasma is not visible. Inferiorly, the structure extends into the pituitary fossa, compressing and displacing the pituitary gland against the floor. Posteriorly, it extends into the interpeduncular cistern, splaying both cerebral peduncles and abutting the mid-brain and mammillary bodies, which are compressed and displaced posteriorly. Superiorly, it effaces the third ventricle, causing proximal obstructive dilatation of the lateral ventricles. Laterally, it compresses the bilateral temporal lobes (right more than left) and abuts the bilateral thalami, causing mild compression (Figures 1-2).

**Figure 3:**
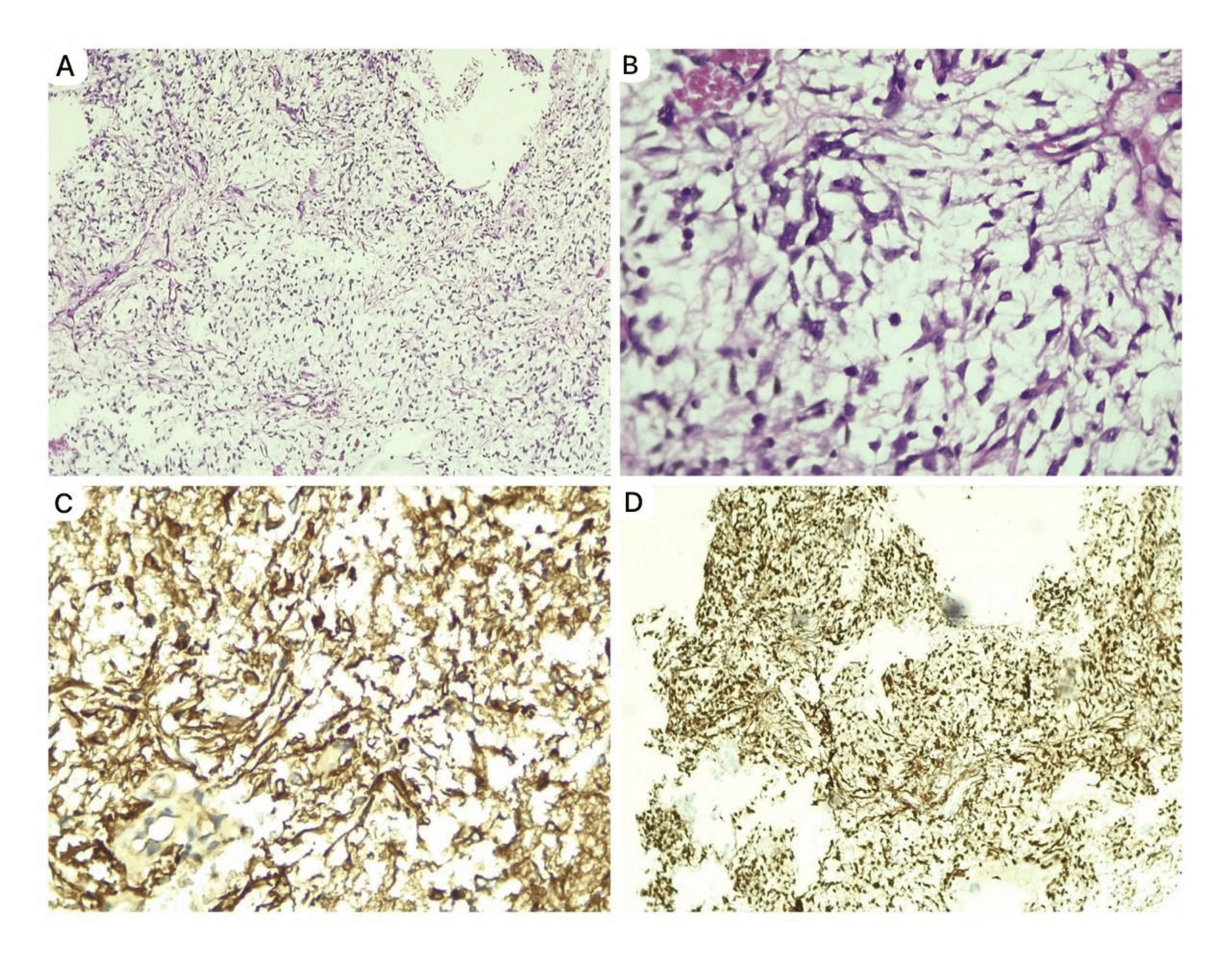
Photomicrograph A) Monomorphous bipolar cells arranged in an angiocentric pattern (H&E, 100x). B) Higher magnification showing glial cells (H&E, 400x). C and D) immunohistochemistry showing positive for markers GFAP and S100, respectively. H&E: Hematoxylin and eosin stain; GFAP: glial fibrillary acidic protein

**Figure 4:**
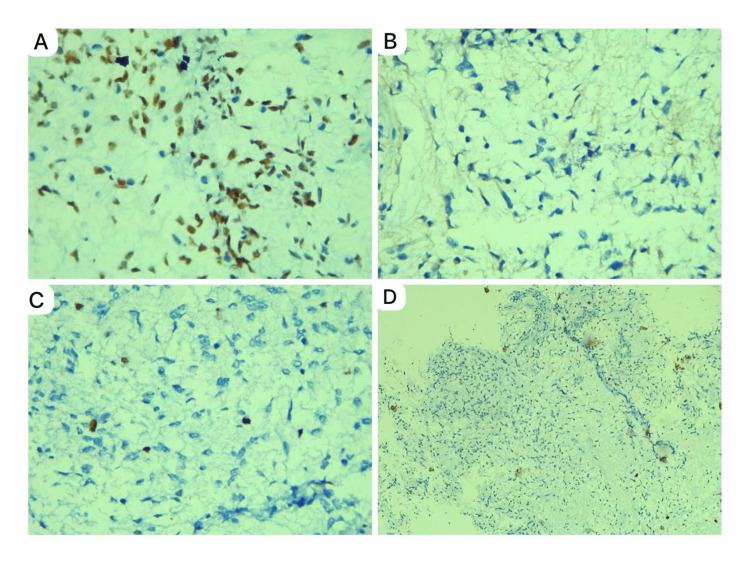
Photomicrograph showing immunohistochemistry A) Retained ATRX, 400x. B) EMA negative, 400x. C) KI 67 3-4%, 400x. D) IDH1 negative, 100x. EMA: epithelial membrane antigen; IDH: isocitrate dehydrogenase

To rule out possible endocrine disturbances, serum cortisol and thyroid-stimulating hormone (TSH) levels were measured, which came back within normal limits at 10 mcg/dL (3.70-19.40 mcg/dL) and 1.11 mIU/L (0.70-6 mIU/L), respectively. The cerebrospinal fluid (CSF) analysis was negative for malignant cells. A neuroendoscopic biopsy with septostomy was done, which confirmed a neoplasm composed of monomorphous bipolar cells and spindle cells predominantly in an angiocentric pattern. The background was myxoid, with focal areas of vascular proliferation in the form of linear glomeruloid tufts. There was no evidence of Rosenthal fibers, eosinophilic granular bodies, mitosis, necrosis, or cystic changes. The histomorphological features were consistent with PMA. Immunohistochemistry showed diffuse positivity for markers S100 and glial fibrillary acidic protein (GFAP) (Figure 3) and negativity for isocitrate dehydrogenase 1 (IDH1) and epithelial membrane antigen (EMA). The Ki67 proliferation index was 3%-4%, and ATRX expression was retained (Figure 4).

Due to obstructive hydrocephalus, the child underwent ventriculoperitoneal shunt placement, resulting in improved muscle tone, strength, and speech. The preferred treatment is a surgical resection. This option was offered to the child's parents, but due to the tumor's deep-seated location and potential long-term neurological sequelae, they decided against surgery. The child was referred to an oncologist, underwent a cycle of radiotherapy, and is currently under follow-up with the oncologist.

## Discussion

PMA can arise throughout the neuraxis, though it often occurs in the hypothalamic region, as in our case, where it was located in the suprasellar region. It primarily affects young children, typically diagnosed around 18 months of age. Common symptoms include increased intracranial pressure or compression of brain tissue, resulting in failure to thrive, altered consciousness, developmental delay, vomiting, feeding difficulties, and generalized weakness. Gait abnormalities, dysmetria, and nystagmus are also frequently observed. Occasionally, tumor extension into the foramen magnum can cause neck stiffness or head tilt, and strabismus due to sixth nerve paresis is less common. Infants may initially present with an increased head circumference. Elevated intracranial pressure can lead to split sutures and a bulging fontanelle. As the condition progresses, affected children may exhibit irritability, lethargy, bradycardia, and slowed respiration. PMA may also cause focal symptoms such as visual disturbances and hypothalamic dysfunction [4]. In our case, the child exhibited a regression of previously attained developmental milestones, weakness in all four limbs, downward gaze, and obstruction of CSF flow at the level of the third ventricle. There were no signs of hormonal or visual disturbances. Despite irritability, the child's sensorium remained preserved.

PMA typically appears on MRI with a hypointense signal on T1-weighted imaging, hyperintensity on T2-weighted imaging and fluid-attenuated inversion recovery (FLAIR) sequences, and a solid structure with central necrosis. Calcifications are rare, occurring in less than 10% of cases, and some cases may show intertumoral hemorrhage. Contrast enhancement varies, and there is usually no diffusion restriction on diffusion-weighted imaging (DWI) in most cases. On computed tomography (CT) imaging, PMA appears as hypodense lesions [5]. In our case, the radiological findings were consistent with these characteristics. There was contrast enhancement at the peripheries, with a few nodular solid-enhancing components. With no diffusion restriction, susceptibility-weighted imaging showed a signal dropout suggesting hemorrhage, and no calcifications were present.

PMA is distinguished by a consistent spread of bipolar spindle cells in a loose myxoid stroma, highlighted by Alcian blue staining. Mitoses are rarely observed. The tumor cells typically form angiocentric pseudo-rosettes and may invade the surrounding neuropil. Unlike PA, which has a biphasic structure alternating with dense cellular areas and loose cystic regions, PMA lacks this biphasic pattern. Rosenthal fibers, eosinophilic granular bodies, and calcifications are generally absent. Microvascular changes, such as telangiectatic clusters in the cyst wall and necrosis without pseudo-palisading, can also be noted [6]. These findings are consistent with our case. In PMA, immunohistochemical staining shows strong and diffuse reactivity to GFAP and S100. GFAP is a primary component of astrocyte intermediate filaments and a marker for reactive astrocytes [7]. Ki-67, a marker used for grading astrocytoma and predicting prognosis, indicates tumor growth quantitatively. A low Ki-67 index suggests a low-grade tumor. In our case, both S100 and GFAP are positive, with a Ki-67 index of 3%-4%, indicating a low-grade tumor [8]. PA are negative for IDH1 mutations and retain ATRX nuclear staining, indicating the absence of ATRX mutations. In contrast, diffuse astrocytoma and anaplastic astrocytoma are positive for IDH1 mutations and show loss of ATRX nuclear staining [9]. In our case, PMA is also negative for IDH1 mutations and retains ATRX nuclear staining. According to the recent WHO Classification of Tumors, PMAs are classified as pediatric-type diffuse low-grade gliomas. This category includes diffuse astrocytoma, angiocentric glioma, polymorphous low-grade neuroepithelial tumor of the young, and diffuse low-grade glioma [3]. Similar to PA, PMA exhibits a simple genomic structure with few alterations. The primary pathways affected are mitogen-activated protein kinase (MAPK) and fibroblast growth factor receptors (FGFR). BRAF fusion and tandem duplications, activating the MAPK pathway, are the most common changes. Key genes involved include BRAF, NF1, FGFR1, and PTPN11 with other mutations being rare [10]. Molecular typing is necessary for differentiation, but it was not performed in our case. Surgical resection is the primary treatment for astrocytic gliomas, with feasibility depending on tumor location. Tumors in midline structures can sometimes be aggressively resected, leading to long-term disease control. However, such procedures can cause significant neurological problems, especially in children under the age of two years. For pediatric-type diffuse low-grade gliomas in deep-seated areas, extensive resection may not be appropriate, and a biopsy should be considered instead. The treatment options for unresectable low-grade gliomas remain controversial [11]. In our case, surgery was offered as an option to the parents, but they opted against it because of possible neurological sequelae. For individuals presenting with obstructive hydrocephalus, a shunt or another procedure to divert cerebrospinal fluid may also be necessary, which is done in our case. Chemotherapy can shrink tumors, delaying or avoiding radiation in most cases, including adolescents with optic nerve gliomas and children with hypothalamic gliomas. Common regimens for nonresectable pediatric low-grade gliomas include carboplatin with or without vincristine and thioguanine, procarbazine, lomustine, and vincristine. A study by Ater et al. [12] compared two chemotherapy regimens for pediatric low-grade gliomas: carboplatin and vincristine (CV) versus thioguanine, procarbazine, lomustine, and vincristine (TPCV). The five-year overall survival rates were 86% for the CV regimen and 87% for the TPCV regimen. For pilocytic astrocytoma, the overall survival was 87%, while, for hypothalamic/optic chiasmal tumors, it was 79% [12]. Radiation therapy generally provides long-term radiographic disease control for most children with chiasmatic and posterior pathway gliomas. However, visual outcomes remain variable despite successful radiological control [11]. A study by Komotar et al. [13] compared 21 hypothalamic PMA patients with 42 hypothalamic PA patients. PMA showed a higher recurrence rate of 76% compared to 26% in PA, despite similar degrees of gross total resection. Additionally, 14% of PMA cases had cerebrospinal fluid dissemination, which was not observed in PA cases [13].

## Conclusions

We discussed a rare case of PMA in a two-year-old boy with developmental regression. These tumors, though classified as low-grade pediatric gliomas, are more aggressive than PA. Surgical resection is the preferred treatment, offering good disease control but with a risk of recurrence. Treatment for deep-seated, non-resectable tumors is controversial due to potential neurological complications. Chemotherapy can shrink tumors, delay radiotherapy, and treat recurrences, while radiotherapy offers good control in optic chiasma tumors, though visual outcomes vary. However, clear guidelines for treatment are lacking, highlighting the need for standardized protocols.
